# ADAM12 expression is upregulated in cancer cells upon radiation and constitutes a prognostic factor in rectal cancer patients following radiotherapy

**DOI:** 10.1038/s41417-023-00643-w

**Published:** 2023-07-26

**Authors:** Krzysztof Bartłomiej Piotrowski, Laia Puig Blasco, Jacob Samsøe-Petersen, Rikke Løvendahl Eefsen, Martin Illemann, Victor Oginga Oria, Karla Iveth Aguilera Campos, Alexia Mélanie Lopresti, Reidar Albrechtsen, Claus Storgaard Sørensen, Xiao-Feng Sun, Marie Kveiborg, Sebastian Gnosa

**Affiliations:** 1 Biotech Research and Innovation Centre (BRIC), University of Copenhagen, Copenhagen, Denmark; 2 Department of Oncology, Herlev Gentofte Hospital, Herlev, Denmark; 3 Department of Oncology and Department of Biomedical and Clinical Sciences, Linköping University, Linköping, Sweden; 4 Present Address: Minerva Imaging, Lyshøjvej 21, Ølstykke, Denmark

**Keywords:** Biomarkers, Cancer

## Abstract

Radiotherapy is one of the most common cancer treatments, yet, some patients require high doses to respond. Therefore, the development of new strategies leans toward personalizing therapy to avoid unnecessary burden on cancer patients. This approach prevents the administration of ineffective treatments or uses combination strategies to increase the sensitivity of cancer cells. ADAM12 has been shown to be upregulated in many cancers and correlate with poor survival and chemoresistance, thus making it a potential candidate responsible for radioresistance. Here, we show that ADAM12 expression is upregulated in response to irradiation in both mouse and human cancer cells in vitro, as well as in tumor tissues from rectal cancer patients. Interestingly, the expression of ADAM12 following radiotherapy correlates with the initial disease stage and predicts the response of rectal cancer patients to the treatment. While we found no cell-autonomous effects of ADAM12 on the response of colon cancer cells to irradiation in vitro, depletion of ADAM12 expression markedly reduced the tumor growth of irradiated cancer cells when subcutaneously transplanted in syngeneic mice. Interestingly, loss of cancer cell-derived ADAM12 expression increased the number of CD31^+^FAP^−^ cells in murine tumors. Moreover, conditioned medium from ADAM12^−/−^ colon cancer cells led to increased tube formation when added to endothelial cell cultures. Thus, it is tempting to speculate that altered tumor vascularity may be implicated in the observed effect of ADAM12 on response to radiotherapy in rectal cancer. We conclude that ADAM12 represents a promising prognostic factor for stratification of rectal cancer patients receiving radiotherapy and suggest that targeting ADAM12 in combination with radiotherapy could potentially improve the treatment response.

## Introduction

Treatment options for cancer have significantly advanced during the past decades and current regimens have enhanced both patient outcome and quality of life. About 50% of all cancer patients receive ionizing radiation, which makes radiotherapy (RT) a major treatment modality. Technological developments have lowered the quantity of cytotoxic radiation deposited in healthy tissues, and therefore unfavorable side effects, while increasing the dose administered in the tumor [1]. However, many patients fail to respond to RT due to resistance of the tumor [2]. It is, therefore, essential to find biomarkers to stratify patients, who will likely benefit from the RT, and to identify novel drug targets that can sensitize the tumor to irradiation.

Radioresistant cancer cells often show alterations in genes involved in DNA repair, cell cycle control and apoptosis [3]. Additionally, cancer cells have the ability to create a radioresistant tumor microenvironment (TME) by altering angiogenesis, modifying the extracellular matrix and interacting with infiltrating immune cells [4]. The communication of cancer cells with cells of the TME is mediated by paracrine signaling molecules such as growth factors and cytokines and their cognate cellular receptors [5, 6]. Importantly, many of these signaling molecules are shed from the cell surface by a disintegrin and metalloproteinases (ADAMs), placing these enzymes as key regulators of cell–cell communication [7, 8].

ADAMs constitute a family of 21 transmembrane glycoproteins [9], of which approximately half have a functional metalloprotease domain. Each of the proteolytically active ADAMs typically sheds multiple substrates, and ADAM-mediated shedding can occur in both a constitutive and an inducible manner, depending on the ADAM and the specific substrate [10]. Some of the ADAMs, such as ADAM9, ADAM10, ADAM12, ADAM15, and ADAM17, are frequently upregulated in tumor tissues as well as cancer cell lines, and their expression often correlates with adverse patient survival and/or treatment response [11–17].

Interestingly, it has been previously shown that ADAM10 and ADAM17 expression and enzymatic activity are induced by irradiation and that the depletion of these proteases radiosensitize tumor cells [18–20]. However, attempts to block these molecules have failed so far, mainly due to lack of specificity of the inhibitory molecules and an incomplete knowledge about the complex functions of ADAM10 and ADAM17, including how they mediate the crosstalk between cells in the TME [21–23]. ADAM12, on the other hand, has not been studied in the context of RT. Yet, it is almost exclusively expressed in the neoplastic cells in human tumors and therefore represents an attractive therapeutic cancer target [17, 24]. We and others have shown that ADAM12 is upregulated in multiple types of cancer and that its expression correlates with worse patient survival [17, 25, 26]. Moreover, ADAM12 promotes tumor progression in multiple mouse models [17, 24, 27, 28] and its expression has been linked to chemoresistance [29]. Mechanistically, ADAM12 regulates cancer cell proliferation and survival, either through its ability to shed growth factors and adhesion molecules from the surface of tumor cells or via its interaction with cell surface molecules such as integrins [25, 30]. Given these findings, we aimed to investigate the role of ADAM12 in the response of rectal cancer to RT.

## Materials and methods

### Patient material

Paraffin embedded tissue microarrays (TMAs) containing samples from 158 rectal cancer patients, including 120 distant normal mucosa (109 of which were taken from the same patients as the primary tumors), 146 resected primary rectal adenocarcinomas (primary tumor) and 49 lymph node metastases (44 of which were taken from the same patients as the primary tumors) were analyzed.

The patients were from the South-East Swedish Health Care region and were enrolled in the randomized Swedish rectal cancer trial of preoperative RT between 1987 and 1990 [31]. Among the 158 patients, 83 received surgery alone (non-RT), while 75 patients received preoperative RT. The total dose administered was 5×5Gy, given within 7 days (range 4–12). None of the patients received adjuvant therapy. The surgery was performed after a median of 3 days (range 0–11 days). The mean follow-up period was 83 months (range 0–193 months), and 54 patients died from the cancer. The median patient age was 69 years (range 36–85 years). More patient and tumor characteristics are presented in Table 1.

**Table 1:** ADAM12 expression in primary rectal tumors in relation to clinicopathological variables.

	Surgery			RT and surgery		
Variables	ADAM12 expression			ADAM12 expression		
	Weak (%)	Strong (%)	*p*-Value	Weak (%)	Strong (%)	*p*-Value
*Gender*			0.69			0.18
Male	16 (38)	26 (62)		9 (23)	30 (77)	
Female	19 (59)	13 (41)		9 (39)	14 (61)	
*Age, years*			0.24			0.2
≤69	20 (54)	17 (46)		13 (35)	24 (65)	
>69	15 (36)	27 (64)		5 (20)	20 (80)	
*Tumor stage*			0.67			**0.001**
I	11 (55)	9 (45)		11 (61)	7 (39)	
II	10 (48)	11 (52)		5 (20)	20 (80)	
III	14 (42)	19 (58)		2 (11)	17 (89)	
*Differentiation*			0.96			0.21
Better	23 (49)	24 (51)		12 (29)	29 (71)	
Worse	4 (50)	4 (50)		0 (0)	4 (100)	
*Local recurrence*			0.41			0.19
No	28 (50)	28 (50)		18 (31)	40 (69)	
Yes	7 (39)	11 (61)		0 (0)	4 (100)	
*Distant recurrence*			0.71			**0.003**
No	23 (49)	24 (51)		17 (41)	24 (59)	
Yes	12 (31)	27 (69)		1 (5)	20 (95)	

Statistically significant *p*-values are shown in bold.

### Immunohistochemistry

Three micrometre thick paraffin sections were cut from the formalin fixed paraffin embedded TMAs and used for immunohistochemistry (IHC) staining. Antigen retrieval was performed by an enzymatic treatment with Proteinase K (5 µg/µl) in a proteinase K buffer (50 mmol/L Tris-HCl, 50 mmol/L EDTA, pH 8.0) at 37 °C for 10 min. Endogenous peroxidase activity was blocked by incubation with 1% H_2_O_2_ for 15 min. The sections were washed in Tris-buffered saline (TBS, 50 mM Tris-HCl, 150 mM NaCl) containing 0.5% Triton X-100 (TBS-T) and then mounted in Shandon racks with immunostaining cover plates (Thermo Scientific) for further incubations. The polyclonal anti-ADAM12 rabbit serum (rb122), which has been previously described [32], was diluted 1:150 in Antibody Diluent with Background-Reducing Components (Dako, id: S3022), added to the slides, and incubated overnight at 4 °C. The polyclonal antibodies were detected with EnVision Horseradish Peroxidase Rabbit and each incubation step was followed by washes in TBS-T. The sections were developed with NovaRed (Vector laboratories) and counterstained in Mayer’s hematoxylin. Finally, slides were dehydrated and mounted with Pertex.

The stained TMAs were scanned and scored semi-quantitatively by three independent observers, including a clinical pathologist, in a blinded fashion. Based on the intensity of staining, the samples were scored as negative, weak, moderate, or strong staining. For statistical analysis, negative and weak-stained samples were considered as low-expressing, and moderate and strong staining as high-expressing. Chi-square test or Fisher exact *t*-test was applied to test for significantly different ADAM12 expression between the samples and clinicopathological variables. Log-rank test was used to evaluate the differences in survival.

### Cell culture

All cell lines were obtained from American Type Culture Collection (ATCC). CT26 cells (id: CRL-2638) were cultured in RPMI 1640 medium supplemented with 100U/ml penicillin and 100 µg/ml streptomycin (all Gibco) and 10% FBS (GE Healthcare). SW480 (id: CCL-228), MDA-MB-231 (id: CRM-HTB-26) and MC38 (kind gift from Dr. Daniel Madsen, Herlev Hospital) cells were maintained in DMEM medium supplemented with 100U/ml penicillin and 100 µg/ml streptomycin (all Gibco) and 10% FBS (GE Healthcare). MC38 cells were additionally supplemented with 1 mM Sodium Pyruvate (Thermo Scientific), MEM Non-Essential Amino Acids Solution and 10 mM HEPES (both Gibco). HUVEC (id: CRL-1730) were cultured in endothelial cell growth medium supplemented with 2% fetal bovine serum, 0.4% endothelial cell growth supplement, 100 pg/ml human recombinant epidermal growth factor, 1 ng/mL human basic fibroblast growth factor, 90 µg/mL heparin and 1 µg/ml hydrocortisone (all from PromoCell). All cells were maintained in a humidified incubator at 37 °C, 5% CO_2_, regularly harvested at 80% confluence to maintain exponential growth and regularly tested for mycoplasma infection.

### Ionizing radiation

Cells were irradiated using the Faxitron CP-160 X-ray irradiation machine (Faxitron Bioptics). The dose rate of ionizing radiation was 1 Gy/min. Dose was adjusted by selecting the corresponding irradiation time and is specified for every experiment.

### Analysis of gene expression upon irradiation

CT26 cells were seeded at a density of 7.5 × 10^4^ cells/well in a 6-well plate, incubated overnight, and irradiated with 5 Gy or 0 Gy. MDA-MB-231 and SW480 cells were seeded at a density of 4 × 10^5^ cells/well in a 6-well plate, incubated overnight, and irradiated with 10 G or 0 Gy. Next, cells were maintained for the indicated time periods and total RNA was purified using RNeasy Mini Kit (Qiagen) according to the supplier’s protocol. cDNA was obtained by reverse transcription of 400 ng of previously purified RNA and performed using RevertAid Reverse Transcriptase with random hexamer primers (both Thermo Scientific), according to the manufacturer’s guidelines. Quantitative PCR (qPCR) was performed using Maxima SYBR Green/ROX Master Mix (Thermo Scientific). qPCR primers are listed in supplementary table 2. Gene expression was normalized to at least two of the following housekeeping genes: *GAPDH*, *RPL13a*, *B2m* for mouse and *GAPDH*, *ACT3*, *RPL9* for human. Fold change of mRNA expression was calculated by comparing the irradiated sample to its corresponding non-irradiated control at each time point. The average of three technical replicates was used to calculate each biological replicate.

### Analysis of cell surface ADAM12 levels using flow cytometry

CT26 cells were seeded at a density of 3 × 10^5^ cells/dish in 6 cm dishes, incubated overnight, and irradiated with 5 Gy 24 h before the analysis of cell surface ADAM12 levels by flow cytometry. Next, cells were detached from the dish by extensive washing, resuspended in 4 °C PBS with 5% FBS, and filtered through a 50 µm strainer to ensure single cell suspension. To block non-specific binding, cells were resuspended in 300 µl PBS with 5% FBS and 5 µl TruStain FcX Antibody (Biologend, id: 101319) and incubated for 15 minutes at 4 °C. Afterwards, cells were resuspended in 200 µl PBS with 5% FBS and incubated with rb122 ADAM12 antibody or pre-immunized serum [32], using a 1:300 dilution in PBS with 5% FBS for 30 minutes at room temperature. Next, cells were stained with anti-rabbit Alexa Fluor 546 antibody (ThermoFisher, id: A10040) at dilution 1:200 in PBS with 5% FBS. Flow cytometry was performed using a FACSAria III Cell Sorter (BD Bioscience).

### Generating ADAM12-knockout cells using CRISPR/Cas-9 gene editing

SW480, MDA-MB-231 and CT26 ADAM12-knockout cells were developed using the previously described CRISPR/Cas-9 system [33]. Single-guide RNAs (sgRNAs) were selected using the WTSI genome editing website [34]. After testing for sgRNAs efficiencies, we chose sgRNAs 5′-CCCGCATTTGAGAGGTTCCA-3′ for murine CT26 cells and 5′-CTCCCTCGCTCGAAATTACACG-3′ for human SW480 and MDA-MB-231 cells. Next, they were inserted into the pSpCas9(BB)-2A-GFP vector as previously described [33]. After transfection, GFP-positive cells were single-cell sorted. ADAM12-knockout was verified using qPCR, Indel Detection by Amplicon Analysis (IDAA) [35] and tested for bi-allelic frameshift using Sanger sequencing (Eurofins).

### Development of cells stably overexpressing ADAM12

A vector containing the murine ADAM12 and mCherry expression constructs flanked by sequences recognized by the sleeping beauty transposase was purchased from VectorBuilder (id: VB200825-1117fur). Point mutation E359Q was introduced using whole genome PCR and confirmed by Sanger sequencing (Eurofins). Plasmids were transfected with the sleeping beauty transposase into MC38 cells using Lipofectamine 3000 and into CT26 cells using electroporation. 48 h after transfection mCherry-expressing cells were sorted using FACS Aria III Cell Sorter (BD Bioscience).

### Western blot

Cells were lysed using RIPA buffer (50 mM Tris-HCl pH 7.5, 150 mM NaCl, 1 mM, EDTA, 0.1% SDS, 1% Triton X-100, 0.5% Sodium Deoxycholate), supplemented with 10 µM Batimastat and 10 mM 1.10 Phenanthroline (both Sigma-Aldrich). Protein concentration was determined using BCA protein assay reagent kit (Thermo Scientific). Normalized protein amounts mixed with 4× Laemmli buffer were denatured at 95 °C for 5 min, separated by 10% SDS-PAGE and blotted onto PVDF membranes (Merck). For the blocking and the antibodies suspension, 5% milk (Sigma-Aldrich) in TBS-T was used. Membranes were blocked for 1 h at room temperature. Next, they were incubated with the primary antibody for 12 h at 4 °C, washed, and incubated with horseradish peroxidase-conjugated secondary antibody for 1 h at room temperature. Finally, they were developed using ECL detection solution (GE-Healthcare) and images were taken using ImageQuant LAS 4000 (GE-Healthcare). The primary and secondary antibodies used were: rabbit anti-ADAM12 rb122 (1:1000, as described [32]), rabbit anti-ß-actin (1:1000, Cell Signaling Technology, id: 4967), donkey anti-rabbit-HRP (1:2000, GE-Healthcare, id: NA934).

### AP-EGF shedding assay

MC38 cells overexpressing negative control (NC), ADAM12 wildtype (A12) or catalytically inactive ADAM12 (A12^E359Q^) were seeded at a density of 60 × 10^3^ cells/well in 12-well plates, incubated overnight, and transiently transfected with AP-EGF plasmid (kindly provided by Dr. S. Higashiyama, Ehime University Graduate School of Medicine, Ehime, Japan), using Lipofectamine 3000 and following the supplier’s protocol. After 24 h incubation, cells were washed and 450 µl of DMEM without FBS was added to each well and incubated for 1 h. Collected media were spun down at 400 g for 5 minutes and supernatants transferred to new tubes to ensure no cell carryover. Cells growing in monolayers in 12-well plates were lysed using RIPA buffer supplemented with 1× HALT Protease and Phosphatase inhibitor cocktail (Thermo Scientific). Abundance of alkaline phosphatase in conditioned medium or cell lysates was quantified by adding a 2 mg/ml solution of SIGMAFAST p-Nitrophenyl phosphate (Sigma-Aldrich), incubating the samples for 1 h at 37 °C, and measuring absorbance at 405 nm. Relative EGF shedding was quantified using the formula S = C/(L + C) where S—relative shed EGF, C—quantity of AP in conditioned medium, and L—quantity of AP in the cell lysate.

### Clonogenic assay

Depending on the dose used for irradiation, 300–1200 cells/well were seeded in each well of 6-well plate and immediately irradiated. Next, MC38 and CT26 cells were incubated for 7 days and MDA-MB-231 and SW480 cells were incubated for 12 days. Afterwards, the cells were fixed using ice-cold methanol for 6 min and stained using 0.5% crystal violet in 20% methanol diluted in water for 20 min. Fixed and stained cells were washed 3 times with water, dried, and manually counted. Aggregate of at least 50 cells was counted as a colony. Plating efficiency and surviving fraction of cells were counted using the formula described by Franken et al. [36]. To test for significant differences in the cells’ radiosensitivity, their response to ionizing radiation was modeled using a linear-quadratic cell death model and compared using extra sum-of-squares *F*-test using GraphPad (v.9.3.1).

### Angiogenesis assay

In vitro angiogenesis was examined using the tube formation assay as previously described [37]. Briefly, wildtype (WT) or ADAM12 knockout (A12^−/−^) SW480 colon cancer cells were seeded at a density of 10^5^ cells/well in 6-well plates, incubated overnight, irradiated with 0 Gy or 5 Gy and incubated for 48 h. Next, cells were carefully washed 3 times and 1 ml of fresh fetal bovine serum (FBS)-free medium/per well was added and conditioned overnight. Conditioned medium (CM) was collected, centrifuged to remove any cells or cell debris, and filtered through a 0.2 μm filter. Ice-cold flat-bottom 96-well plate was coated with 50 μl Matrigel using the in vitro angiogenesis kit (Sigma-Aldrich, id: ECM625). HUVECs (2 × 10^4^ cells/well) were seeded in 200 μl CM and the plate was incubated for 6 h. At least three technical replicates were made for each condition. Serum-free medium (SFM) was used as an NC, and 0.5 μg/ml human recombinant vascular endothelial growth factor (VEGF) (R&D Systems) in SFM as a positive control. Nine images/condition were obtained using a light microscope and the numbers of branches, junctions, and meshes were counted in a blinded manner using ImageJ, as previously described [37].

### Syngeneic colon cancer mouse model

On the day of injection, CT26 WT or CT26 ADAM12^−/−^ (clone #2) were irradiated with 0 Gy or 5 Gy and immediately harvested. Next, 5 × 10^5^ cells in 100 µl PBS were subcutaneously injected in the flank of 8-week-old male BALB/c mice (Janvier Labs). All animals were kept in individually-ventilated cages in a climate-controlled room at a temperature of 22 ± 2 °C, humidity of 50 ± 5%, 12 h light/dark cycle, and fed a standard diet and water ad libitum. The tumors were measured in a blinded fashion using electric calipers every other day and tumor volume was calculated using the formula: *v* = (*l* × w^2^)/2 where *v*—volume, *l*—length, *w*—width. Comparison of tumor growth was performed by modeling exponential growth curves for each group and comparing models using extra sum-of-squares *F*-test in GraphPad (v.9.3.1). Tumors were dissected from mice upon reaching the size endpoint (*l* ≥ 15 mm or *l* + *w* ≥ 24 mm), fixed in formalin and paraffin embedded. Tumor sections were analyzed by IHC using the anti-CD31 primary antibody (Cell Signaling, id: 77699) diluted 1:100 in phosphate-buffered saline (PBS) with 2.5% normal goat serum, according to previously described standard procedures [23]. For each tumor, staining from four representative pictures were quantified in a blinded manner using ImageJ [38]. Weight and wellbeing of mice were monitored throughout the whole experiment, and all experiments were performed in accordance with authorization and guidance from the Danish Inspectorate for Animal Experimentation (license number: 2019-15-0201-01642).

### Flow cytometry

Nineteen days post-injection, tumors were dissected and immediately placed on ice for flow cytometry-based profiling. The tumors were cut into small pieces, placed in 1 ml digestion buffer containing 2.1 mg/ml collagenase type I (Worthington, BioNordica) and 75 μg/ml deoxyribonuclease type I (Worthinton, BioNordica), and incubated at 37 °C with gentle shaking for 1 h. The digested tumors were then filtered through a 70-μm mesh and lysed using red blood cell lysis buffer (Qiagen) for 2 min at room temperature (RT). The cell suspensions were stained with Zombie Aqua (BioLegend) for 15 min at RT for live/dead exclusion and subjected to Fc receptor blocking (ThermoFisher Scientific) for 10 min at 4 °C. Next, cell suspensions were stained for 15 min at 4 °C using primary fluorescently labeled anti-mouse antibodies to discriminate live and dead cells (#23102, BioLegend, diluted 1:100), and cells expressing the hematopoietic marker CD45 (BioLegend #3011F, diluted 1:200), the fibroblast marker fibroblast activated protein (FAP) (R&D #BAF3715, diluted 1:50), and the endothelial cell marker CD31 (BioLegend #102406/390, diluted 1:100). Following primary antibody staining, cell suspensions were incubated with APC-streptavidin secondary antibody (BioLegend #405207) for 10 min at 4 °C. Analysis was performed using BD FACSAria III instrument (BD Biosciences). Positive and negative gates for each marker were set using fluorescence minus one controls in which each fluorophore in turn was excluded. With the use of FlowJo software (v.10.8) the cell populations of interest were gated.

### Statistical analysis

Unless otherwise specified the statistical analysis was as follows. For comparison of the two groups, Welch’s two-sided *t*-test was performed. For three or more groups of paired data repeated measures one-way ANOVA with Geisser–Greenhouse correction was used. For three or more groups of unpaired data, Welch’s one-way ANOVA was used. For all comparisons using ANOVA the correction for multiple comparisons using false discovery rate (FDR) control by Benjamini, Krieger and Yekutieli method was performed. Chi-square or Fisher exact T-test test was applied to test for significantly different ADAM12 expression between the samples and clinicopathological variables. Log-rank test was used to evaluate the differences in survival. All calculations were done using GraphPad (v.9.3.1) or SPSS. *p* ≤ 0.05 was considered significant.

## Results

### ADAM12 expression after RT predicts cancer relapse

**Fig. 1:**
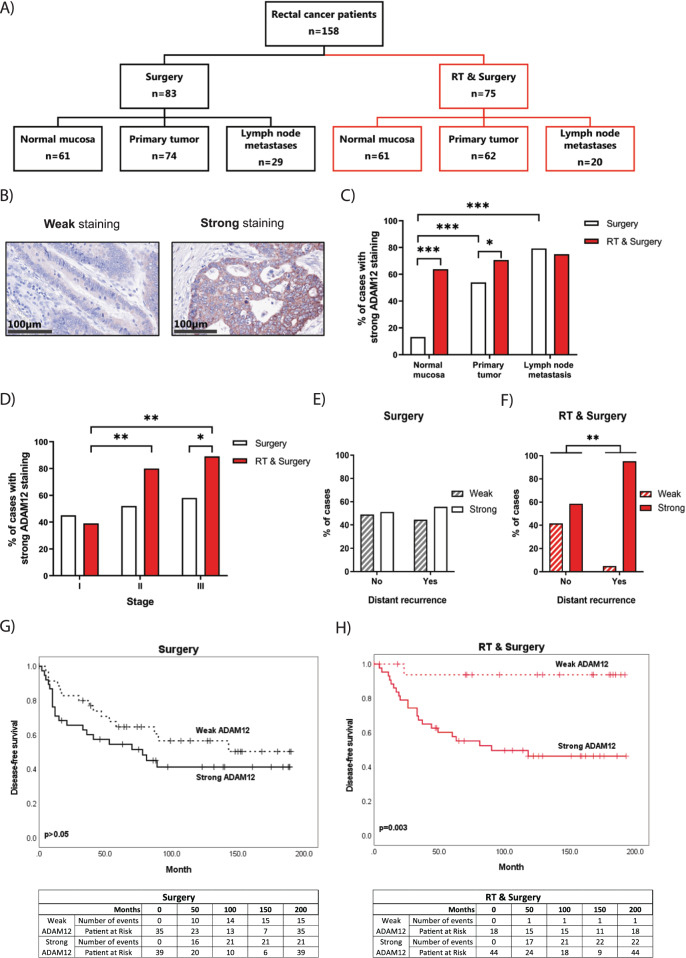
ADAM12 expression is upregulated in patients treated with preoperative radiotherapy and correlates with distant recurrence and disease-free survival. **A** Summary of the patient material included in this study from the Swedish rectal cancer trial of preoperative radiotherapy. **B** Representative IHC images of weak and strong ADAM12 staining in primary tumor samples. **C** Percentage of cases with strong ADAM12 staining in the normal mucosa, primary tumor, and lymph node metastases of the surgery alone and the preoperative radiotherapy plus surgery groups. **D** Percentage of cases with strong ADAM12 staining in stages I–III of the surgery alone and preoperative radiotherapy plus surgery groups. **E**, **F** ADAM12-expressing cases in correlation to distant recurrence in the surgery alone (**E**) and the preoperative radiotherapy plus surgery groups (**F**). **G**, **H** Kaplan–Meier curves for disease-free survival of weak and strong ADAM12-expressing cases in the surgery alone (**G**) and preoperative radiotherapy plus surgery groups (**H**). Associated numbers of events and patients at risk are shown in the tables below. Chi-square (**C**–**F**) or Log-rank (**G**, **H**) test were applied to test for significant differences: *p ≤ 0.05, **p ≤ 0.01, ***p ≤ 0.001.

To investigate the role of ADAM12 in the response of cancer patients to RT, we analyzed ADAM12 expression by IHC in formalin fixed and paraffin embedded samples from rectal cancer patients, who participated in the randomized Swedish rectal cancer trial of preoperative RT [31] (Fig. 1A). We identified either weak or strong ADAM12 staining in all tissue samples and found the expression of ADAM12 to be higher in primary tumors and lymph node metastases in comparison to the normal rectal mucosa (Fig. 1B, C). Interestingly, ADAM12 expression was higher in normal mucosa and primary tumor tissue from patients undergoing RT, as compared to patients undergoing surgery alone. However, the ADAM12 expression was unchanged in lymph node metastases following RT (Fig. 1C). Next, we correlated the expression of ADAM12 with clinicopathological variables of the patients (Table 1). We found that the expression of ADAM12 was significantly higher in late-stage tumors (II and III) compared to the early stage (I), but only in tumors receiving RT (Fig. 1D). Moreover, we found that high ADAM12 expression correlated with a significantly higher risk of developing distant recurrence, but again only in tumors treated with RT (Fig. 1E, F). Interestingly, expression of ADAM12 in the primary tumor did not correlate with disease-free survival in the surgery alone group (Fig. 1G). In contrast, patients with weak ADAM12 expressing tumors had significantly better disease-free survival following RT, as compared to patients with strong ADAM12 expressing tumors (Fig. 1H). While limited by few samples exhibiting low ADAM12 staining, comparison of disease-free survival subdivided into stages I, II, and II indicated that strong ADAM12 expression in especially stage III rectal tumors is a good discriminator for patient survival post-surgery and RT (Supplementary Fig. 1A). Finally, while the assessment of overall survival showed similar results as disease-free survival (Supplementary Fig. 1B), ADAM12 expression did not correlate to local recurrence of the disease (Supplementary Fig. 1C).

### ADAM12 is upregulated in cancer cell lines following irradiation

**Fig. 2:**
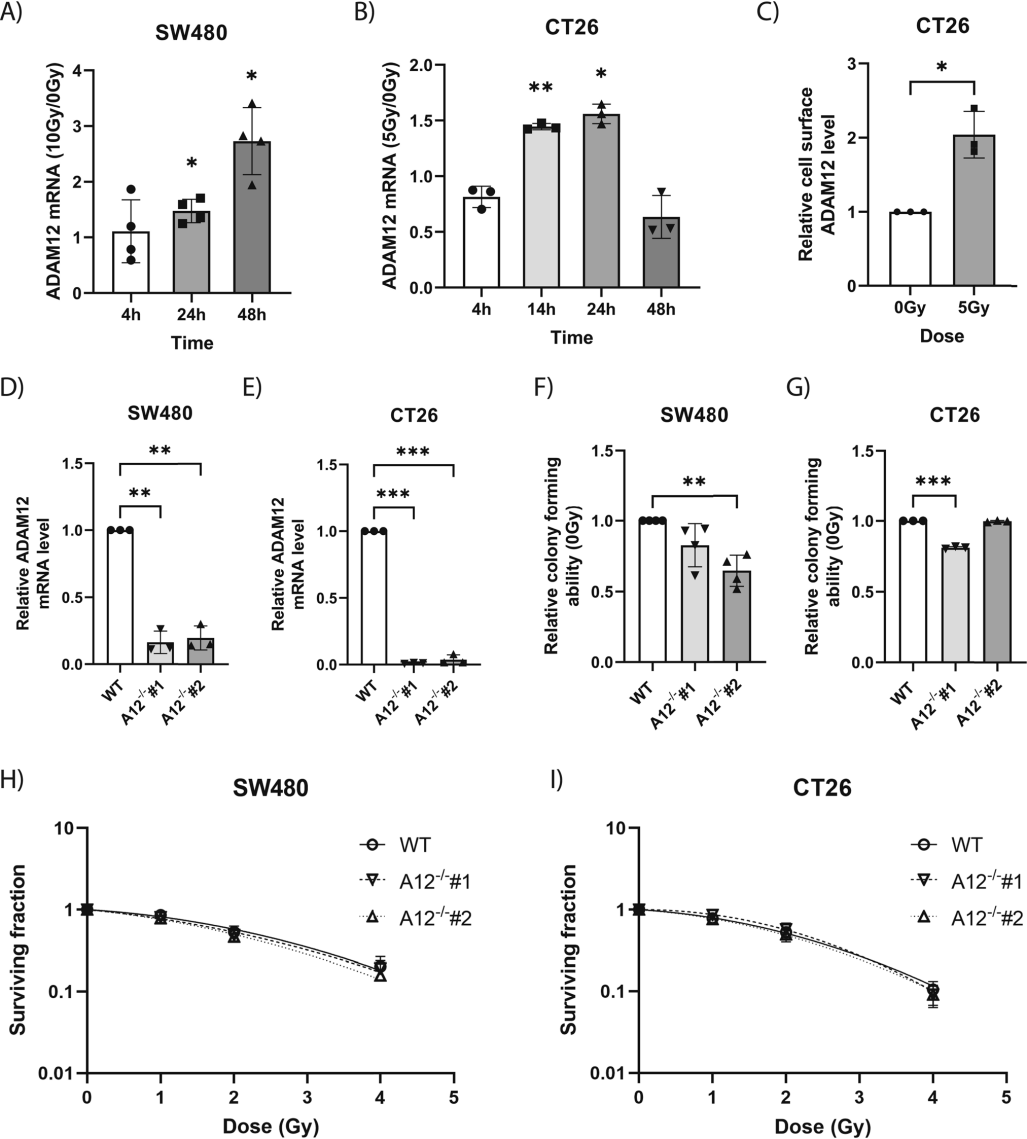
ADAM12 is upregulated in colon cancer cells following irradiation, yet loss of ADAM12 does not affect survival of the cells. **A** Fold change of ADAM12 mRNA levels at different timepoints after irradiation with 10 Gy compared to 0 Gy in SW480 cells, measured by qPCR (*n* = 4). **B** Fold change of ADAM12 mRNA levels at different timepoints after irradiation with 5 Gy compared to 0 Gy in CT26, measured by qPCR. **C** Fold change of cell surface ADAM12 protein levels measured by flow cytometry of CT26 cells 24 h after irradiation with 5 Gy compared to 0 Gy. **D**, **E** Validation of ADAM12-knockout using qPCR in SW480 cells (**D**) and CT26 cells (**E**). **F**, **G** Cells’ relative ability to form colonies following loss of ADAM12 in SW480 (*n* = 4) (**F**) and CT26 cells (**G**). **H**, **I** Clonogenic assay of WT compared to A12^−/−^ following irradiation in SW480 (*n* = 4) (**H**) and CT26 cells (**I**). Repeated measures one-way ANOVA with Geisser–Greenhouse correction and correction for multiple comparisons using FDR control by Benjamini, Krieger and Yekutieli method (**A**, **B**, **D**–**G**), two-sided Welch’s *t*-test (**C**) or extra sum-of-squares *F*-test of linear quadratic cell death models (**H**, **I**) were applied to test for significant differences: *p ≤ 0.05, **p ≤ 0.01, ***p ≤ 0.001. *n* = 3 unless otherwise specified.

To recapitulate the upregulation of ADAM12 expression upon RT observed in rectal cancer patients, we tested the expression levels of ADAM12 in cancer cells following exposure to ionizing radiation in vitro. Analysis of ADAM12 mRNA levels in human SW480 colon cancer cells irradiated with a dose of 10 Gy showed a significant upregulation at both 24 and 48 h post-irradiation (Fig. 2A). Likewise, we observed increased ADAM12 mRNA expression in the murine CT26 colon cancer cell line at 14 and 24 h, following 5 Gy irradiation (Fig. 2B). Additionally, we analyzed whether ADAM12 was upregulated at the protein level following exposure to radiation. We quantified ADAM12 levels at the surface of CT26 cells using flow cytometry and observed an approximately 2-fold increase in ADAM12 surface levels 24 h after irradiation with 5 Gy (Fig. 2C).

We also attempted to induce ADAM12 expression in the murine MC38 colon cancer cell line with ionizing radiation, but our analysis showed that MC38 cells do not express ADAM12 in vitro and we could not induce its expression using ionizing radiation (Supplementary Fig. 2). To test whether the increase in ADAM12 expression following irradiation is limited to colon cancer cell lines, we used the human MDA-MB-231 breast cancer cell line. Here, we observed a more than 1.5-fold upregulation 48 h after irradiation with 10 Gy, indicating a more general effect of ionizing radiation on ADAM12 expression (Supplementary Fig. 3A).

### Loss of ADAM12 does not affect survival of colon cancer cells after irradiation

To test whether radiation-induced ADAM12 increased cell survival upon radiation, we generated *ADAM12*-knockout (A12^−/−^) SW480 and CT26 cell lines and performed clonogenic survival assays.

For each of the cell lines, we confirmed the loss of ADAM12 expression in two independent A12^−/−^ clones (Fig. 2D, E). While, for both SW480 and CT26 cell lines, one of the A12^−/−^ clones showed a reduction in the ability to form colonies (Fig. 2F, G), neither A12^−/−^ SW480 nor A12^−/−^ CT26 cells showed any differences in cell survival following exposure to increasing doses of ionizing radiation, as compared to their non-edited wildtype controls (Fig. 2H, I). We obtained similar results when testing the colony forming capacity and the survival of MDA-MB-231 cells following exposure to ionizing radiation (Supplementary Fig. 3B–D).

### ADAM12 overexpression does not affect survival of colon cancer cells after irradiation

**Fig. 3:**
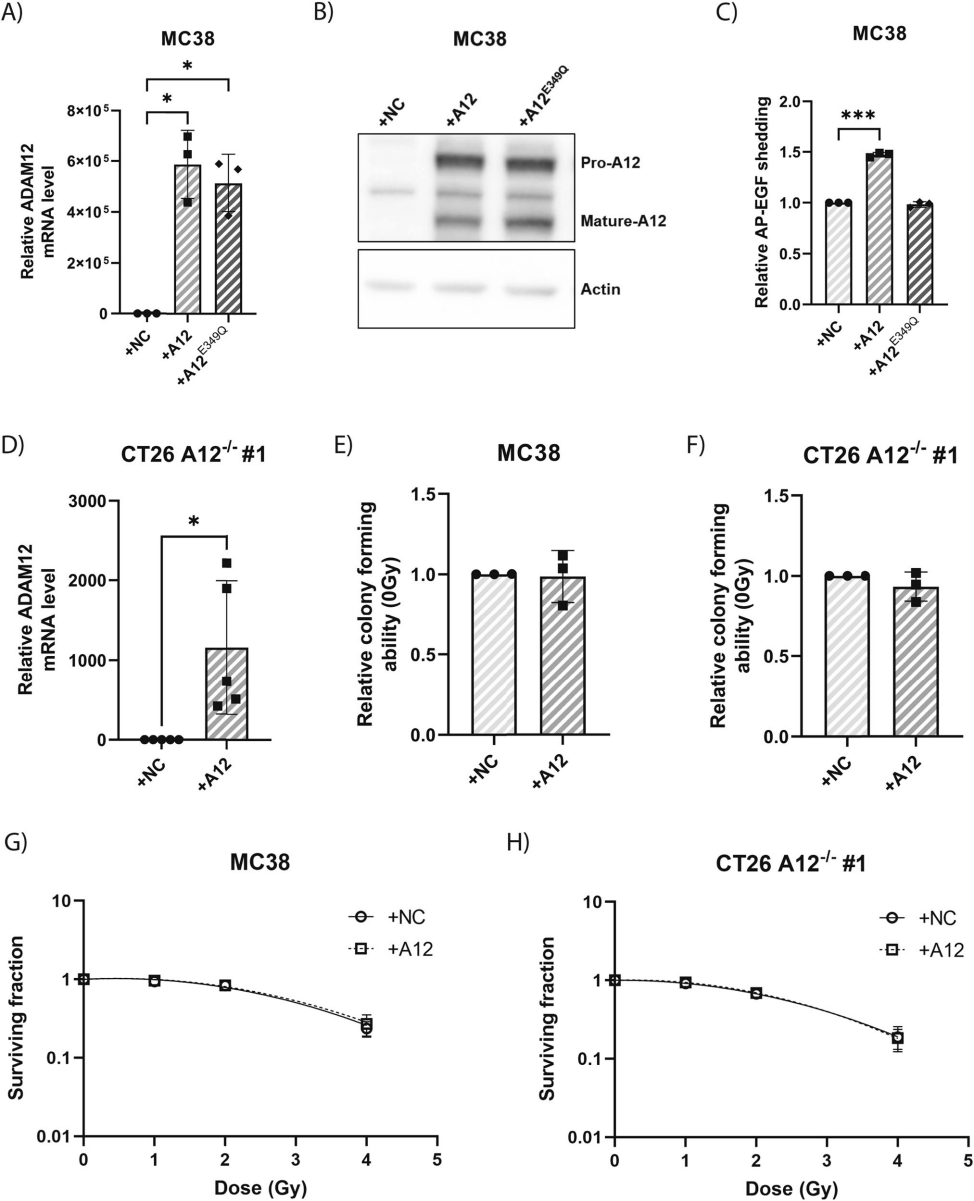
ADAM12 overexpression does not affect colon cancer cell radiosensitivity and ability to form colonies. **A** Validation of overexpression of wildtype ADAM12 (A12) and catalytically inactive ADAM12 mutant (A12^E349Q^) in MC38 cells, as compared to negative control (NC) cells, using qPCR. **B** Western blot confirming overexpression and maturation of ADAM12 in MC38 cells. **C** Confirmation of the catalytic activity of overexpressed A12 compared to cells overexpressing NC or A12^E349Q^, using a cell-based AP-EGF shedding assay. **D** Validation of A12 overexpression in CT26 A12^−/−^ cells (clone #1), as compared to NC cells, using qPCR (*n* = 5). **E**, **F** Relative ability to form colonies in cells overexpressing A12 or NC in MC38 (**E**) and CT26 A12^−/−^ (**F**) cells. **G**, **H** Clonogenic assay of cells overexpressing A12 compared to NC following irradiation in MC38 (**G**) and CT26 A12^−/−^ (**H**) cells. Repeated measures one-way ANOVA with Geisser–Greenhouse correction and correction for multiple comparisons using FDR control by Benjamini, Krieger, and Yekutieli method (**A**, C), two-sided Welch’s *t*-test (**D**–**F**) or extra sum-of-squares *F*-test of linear quadratic cell death models (**G**, **H**) were applied to test for significant differences: *p ≤ 0.05, **p ≤ 0.01, ***p ≤ 0.001. *n* = 3 unless otherwise specified.

To further analyze if the expression of ADAM12 affects the colony forming capacity and survival upon radiation of cancer cells, we overexpressed catalytically active wildtype ADAM12 (A12) in MC38 cells (Fig. 3A). To validate the catalytic activity of the overexpressed A12, we also overexpressed a catalytically inactive ADAM12 mutant (A12^E349Q^) [39]. Analysis of ADAM12 expression using western blot confirmed that overexpression of both A12 and A12^E349Q^ led to expression of both the pro- and mature- forms of ADAM12 in MC38 cells (Fig. 3B). Next, we evaluated the shedding ability of the ADAM12-overexpressing MC38 mutants, using a cell-based alkaline phosphatase-tagged epidermal growth factor (AP-EGF) shedding assay and confirmed an increased shedding upon introduction of A12, but not A12^E349Q^ (Fig. 3C). After confirming that the overexpressed A12 is catalytically active, we also overexpressed it in CT26 A12^−/−^ cells (Fig. 3D) and tested the effect on the capacity of both MC38 and CT26 A12^−/−^ cells to form colonies (Fig. 3E, F). In both cell types, overexpression of A12 did not affect the capacity to form colonies, indicating that the change in colony forming capacity of one of the CT26 A12^−/−^ clones (Fig. 2G) is likely due to clonal variation, rather than a causal consequence of the gene knock-out. Moreover, in both CT26 A12^−/−^ and MC38 cells, the overexpression of ADAM12 did not affect the survival upon radiation (Fig. 3G, H).

In summary, we showed that neither loss nor gain of ADAM12 expression had any effects on the survival to radiation of the tested cancer cell lines in vitro.

### ADAM12 regulates the response to RT in a syngeneic mouse tumor model

**Fig. 4:**
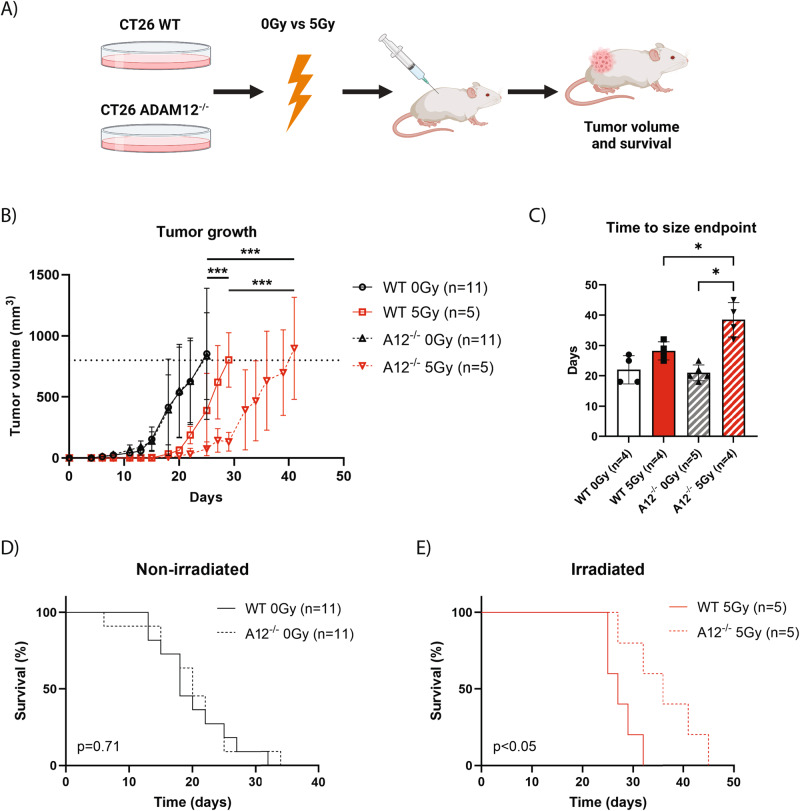
Loss of ADAM12 leads to delayed tumor growth and better survival following IR in a mouse model. **A** Experimental setup of the syngeneic mouse colon cancer model, indicating the irradiation of cells prior to injection. **B** Growth curves of wildtype (WT) and ADAM12-knockout (A12^−/−^) tumors following irradiation of cancer cells with 0 Gy or 5 Gy prior injection. **C** Time required for the different groups to reach the tumor size endpoint. **D**, **E** Kaplan–Meier curves showing the survival of mice bearing WT or A12^−/−^ tumors without irradiation of cancer cells prior to injection (**D**) or following 5 Gy irradiation of cancer cells prior to injection (**E**). Extra sum-of-squares *F*-test of exponential growth models (**B**), Welch’s one-way ANOVA with correction for multiple comparisons using FDR control by Benjamini, Krieger, and Yekutieli method (**C**) or Log-rank test (**D**, **E**) were applied to test for significant differences: *p ≤ 0.05, **p ≤ 0.01, ***p ≤ 0.001.

The lack of effect of ADAM12 on the inherent radiosensitivity of colon cancer cells in vitro, yet a clear irradiation-induced increase in ADAM12 expression and a strong correlation to disease-free survival in patients receiving RT, suggests that ADAM12 may regulate the response to RT via remodeling of the TME. To test this hypothesis, we injected irradiated or non-irradiated WT or A12^−/−^ CT26 cells subcutaneously in the flank of syngeneic mice (Fig. 4A). Interestingly, there were no differences in tumor growth or time required to reach the size endpoint in tumors derived from non-irradiated WT cells versus tumors derived from non-irradiated A12^−/−^ cells. When comparing tumors from non-irradiated versus irradiated cells, growth of both WT and A12^−/−^ irradiated tumors was significantly reduced (Fig. 4B). However, the irradiation-induced delay in tumor growth was significantly stronger in A12^−/−^ tumors as compared to WT tumors, and this effect was further reflected on the time required for the tumors to reach the size endpoint (Fig. 4C).

Importantly, like in rectal cancer patients, there was no effect of ADAM12 expression on mouse survival when comparing non-irradiated WT and A12^−/−^ groups (Fig. 4D). However, following irradiation, A12^−/−^ tumor-bearing mice exhibited improved survival as compared to their irradiated WT counterparts (Fig. 4E).

### Loss of cancer cell-derived ADAM12 expression increases endothelial cell count in CT26 tumors

**Fig. 5:**
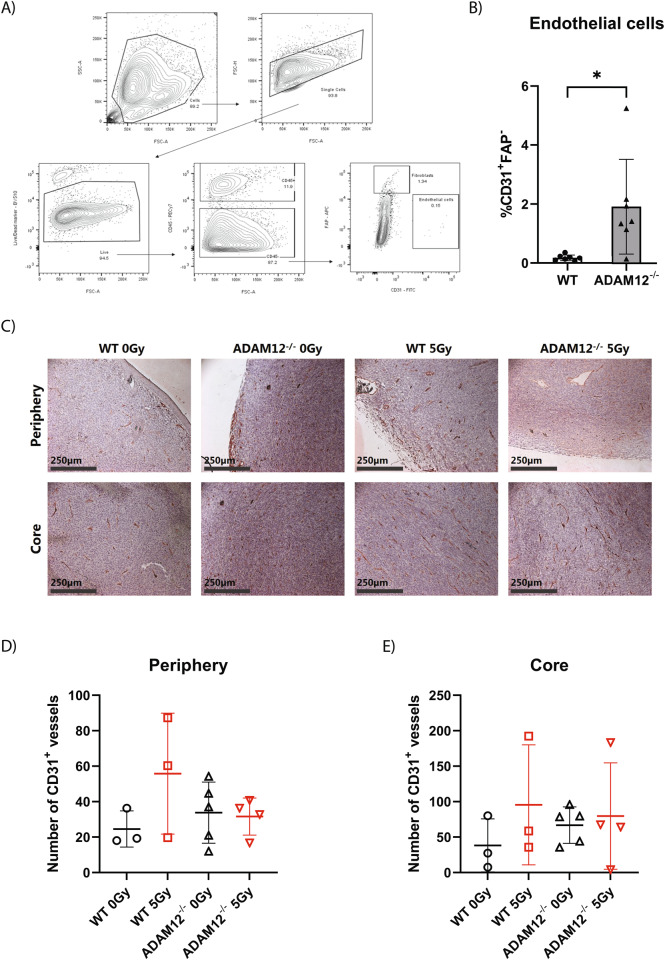
Loss of ADAM12 expression in cancer cells increases endothelial cell count in CT26 tumors. **A** Gating strategy for flow cytometry analysis of dissociated CT26 tumors. First, a size gate was applied to select cells, and another size gate to select single cells, followed by a viability gate (Aqua Zombie negative) to exclude dead cells. Live cells were then gated for CD45 to distinguish hematopoietic from non-hematopoietic cells, and the CD45^−^ cells were further gated for CD31^+^FAP^−^ and CD31^−^FAP^+^ to identify endothelial cells and fibroblasts, respectively. **B** Percentage of CD31^+^FAP^−^ endothelial cells out of live cells in WT versus A12^−/−^ tumors analyzed as shown in (**A**) (*n* = 7). **C** Representative pictures of irradiated (5 Gy) and non-irradiated (0 Gy) WT and A12^−/−^ tumors stained for CD31 by IHC. **D**, **E** Quantification of CD31^+^ vessels per picture in the periphery (**D**) and core (**E**) of non-irradiated (0 Gy) and irradiated (5 Gy) WT and ADAM12^−/−^ tumors. Two-sided Welch’s *t*-test (**B**) or one-way ANOVA with correction for multiple comparisons using false discovery rate (FDR) control by Benjamini, Krieger, and Yekutieli method (**D**, **E**) were applied to test for significant differences: *p < 0.05.

Tumor vascularity greatly influences the response of tumors to radiation treatment [40]. Thus, to investigate the link between ADAM12 expression in cancer cells and in vivo tumor growth following irradiation, we initially compared the number of endothelial cells in WT versus A12^−/−^ tumors. Interestingly, flow cytometry-based quantification of endothelial cells in non-irradiated dissociated tumors showed a marked increase in the number of CD31^+^FAP^−^ cells in ADAM12-deficient tumors (Fig. 5A, B). We next analyzed intact tumors from irradiated and non-irradiated WT versus A12^−/−^ CT26 cancer cells by IHC. While there was a tendency towards an increased number of CD31^+^ vessels in ADAM12-deficient tumors, the differences were not significant in either the periphery of the tumor or the tumor core (Fig. 5C–E).

### ADAM12 regulates the effect of colon cancer cells on neighboring endothelial cells in vitro

**Fig. 6:**
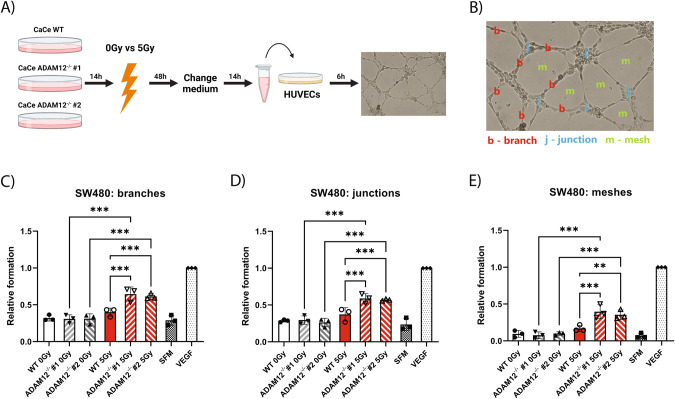
ADAM12 regulates the effect of colon cancer cells on neighboring endothelial cells in vitro. **A** Experimental setup used to obtain conditioned media (CM) from irradiated or non-irradiated WT and A12^−/−^ SW480 colon cancer cells (CaCe) used to test the effect on tube formation in HUVECs (created with BioRender.com). **B** Example of tube formation by HUVECs with examples of branches, junctions, and meshes. **C**–**E** Quantified branches (**C**), junctions (**D**), and meshes (**E**) formed by HUVECs when exposed to CM from SW480 cells, serum-free media (SFM), or recombinant VEGF, relative to stimulation with recombinant VEGF. One-way ANOVA with correction for multiple comparisons using false discovery rate (FDR) control by Benjamini, Krieger, and Yekutieli method was applied to test for significant differences: *p < 0.05, **p < 0.01, ***p < 0.001.

Given the increase in endothelial cells and a tendency toward increased vessel number in A12^−/−^ tumors, we hypothesize that ADAM12 sheds anti-angiogenic factors from the surface of the colorectal cancer cell surface. To test this hypothesis, we treated human umbilical vein endothelial cells (HUVECs) with conditioned media from irradiated and non-irradiated WT and A12^−/−^ SW480 colon cancer cells and assessed the in vitro tube formation (Fig. 6A, B). While we found no significant differences in tube formation when comparing HUVECs treated with media from irradiated versus non-irradiated wildtype SW480 cells, we found a significant increase in the relative formation of all branches, junctions, and meshes when HUVECs were treated with media from irradiated ADAM12-KO SW480 cells as compared to both non-irradiated A12^−/−^ and irradiated WT SW480 cells (Fig. 6C–E).

## Discussion

In this study, we investigated the potential role of ADAM12 in response of cancer to ionizing radiation. Analysis of ADAM12 expression in tissues from 158 rectal cancer patients showed that ADAM12 was upregulated at the protein level in primary tumors compared to normal mucosa. While this has not been reported for rectal cancer before, it aligns with previous observations that ADAM12 expression is increased in different types of cancers [17, 25, 26]. Moreover, we show for the first time in cancer patients that ADAM12 expression is induced by ionizing radiation in both normal and tumor tissues. Interestingly, we did not see any correlation of ADAM12 expression to tumor progression in rectal patients that only underwent surgical removal of the tumor, despite previous reports in some types of cancer [17, 25, 26]. Our observation in non-RT patients is supported by data from the Human Protein Atlas (proteinatlas.org), which shows that ADAM12 expression in colon and rectal adenocarcinomas of stages I-III does not correlate with patient survival [41]. However, we discovered that in patients who received RT, a high ADAM12 expression correlated with tumor stage. Moreover, we showed that ADAM12 expression had a striking effect on disease-free survival and that it correlated with distant recurrence only in patients undergoing RT before surgical removal of the tumor.

Combined, our findings indicate that ADAM12 could be a potential marker for the stratification of patients who should receive RT. It has been shown that loss of ADAM12 decreased breast cancer cell chemoresistance while its overexpression protected cancer cells from treatment [29], which further supports ADAM12’s potential to predict response to treatment. Moreover, ADAM12 levels in serum have recently been linked to patient prognosis in colorectal cancer with an especially strong correlation to metastatic rectal cancer [42]. Likewise, it has been previously shown that ADAM12 urine levels correlate with tumor stage and prognosis in different types of cancer [43–45]. Taken together, this suggests that ADAM12 levels in the serum and urine of rectal cancer patients should be investigated in relation to response to RT.

To unravel the mechanism by which ADAM12 affects tumor progression following RT, we used human SW480, as well as mouse CT26 and MC38 colon carcinoma cell lines, as model systems. Firstly, we validated that ADAM12 expression is upregulated in SW480 and CT26 colon cancer cell lines following irradiation. This mechanism is not limited to colon cancer cells, as we also showed irradiation-induced ADAM12 upregulation in the MDA-MB-231 human breast carcinoma cell line. In line with our findings, others have shown an X-ray-induced upregulation of ADAM12 in human head and neck squamous carcinoma cell lines [46]. Also, γ-ray induces a dose-dependent upregulation of ADAM12 in oral squamous cell carcinoma cells [47].

After confirming that ADAM12 is upregulated by irradiation in colon cancer cell lines similar to tumor tissues in our cohort, we tested whether the loss or gain of ADAM12 affects the cells’ radiosensitivity. We observed no effect on intrinsic radiosensitivity of colon nor breast cancer cells in vitro following either knockout or overexpression of ADAM12. However, this may be cell or cancer-type dependent, as a few previous studies suggested that ADAM12 expression correlated with radiosensitivity in tongue, larynx, and oral cancer cells [46, 47].

In addition to the irradiation-induced upregulation of ADAM12 in both colon cancer cells in vitro and in rectal tumor tissue, our study showed a correlation of ADAM12 expression to patient disease-free survival following RT. Yet, we observed no effect of ADAM12 on the intrinsic radiosensitivity of cancer cells in vitro. Previous findings with other ADAMs have shown that both ADAM10 and ADAM17 are regulated by RT and subsequently affect the cancer response to ionizing radiation [18, 19]. Interestingly, the mechanisms of the ADAM-dependent response of cancer cells to irradiation differ between its family members. The RT induced ADAM17’s proteolytic activity rather than its expression and contributed to non-small lung cancer cells’ radioresistance [18]. On the contrary, ADAM10 expression was induced by ionizing radiation, which led to the remodeling of the TME by ephrinB2 cleavage and resistance of pancreatic cancer to RT [19]. Since ADAM12 mediates cell–cell communication—i.e., by shedding growth factors and cytokines of the cell surface as well as through interactions with integrins [7, 8, 30] - and similarly to ADAM10, ADAM12 expression is induced by ionizing radiation, its indicates that ADAM12 could impact the cancer response to RT by remodeling the TME.

To test the hypothesis that ADAM12 influences the response to RT by remodeling the TME, we injected wildtype or A12^−/−^ CT26 cells, with or without prior irradiation, subcutaneously in the flank of syngeneic mice. In contrast to some mouse studies [17, 24, 27, 28], we observed no significant change in tumor growth or overall mouse survival upon ADAM12 knockout, possibly due to cancer type and its TME specificity. However, when exposing the cancer cells to prior irradiation, we found a striking delay in the growth of A12^−/−^ irradiated tumors as compared to irradiated wild-type tumors. This was further reflected by an improved survival following irradiation of mice bearing A12^−/−^ tumors as compared to their wild-type counterparts.

The lack of difference in survival between non-irradiated wildtype and A12^−/−^ tumors, as well as an improved survival following irradiation, resemble our findings in rectal cancer patients. Thus, our findings indicate that ADAM12 regulates the response of colon cancer cells to RT indirectly by affecting the TME rather than modulating the cell-autonomous radiosensitivity. Vascular endothelial cells constitute an important part of the TME, and tumor vascularization and the associated oxygenation are essential for radiation-induced DNA damage and cell death [40, 48]. Interestingly, we observed an increased number of endothelial cells in A12^−/−^ CT26 tumor transplants, as well as an increased tube formation when treating human endothelial cells with conditioned media from irradiated A12^−/−^ colon cancer cells, as compared to conditioned media from irradiated wildtype cancer cells. Given that these findings could be translated to human rectal cancer patients, it could potentially explain the observed beneficial effects of RT in patients with weak ADAM12 expression.

In summary, we showed for the first time that ADAM12 could be a predictive marker for the stratification of rectal cancer patients before subjecting them to RT. Furthermore, using in vitro and mouse models, we highlighted the need for further studies of ADAM12 as a potential drug candidate to target in combination with RT.

### Supplementary information

Supplementary material

## Data Availability

The data generated in this study are available upon request from the corresponding author.
