# Supplementary material for: ADAM12 expression is upregulated in cancer cells upon radiation and constitutes a prognostic factor in rectal cancer patients following radiotherapy
Source: Cancer Gene Ther. 2023 Jul 26;30(10):1369–81. doi: 10.1038/s41417-023-00643-w (PMC10581903; doi:10.1038/s41417-023-00643-w)
Supplement: Supplementary file 1 — Supplementary material [file 41417_2023_643_MOESM1_ESM.pdf]

Supplementary figure 1

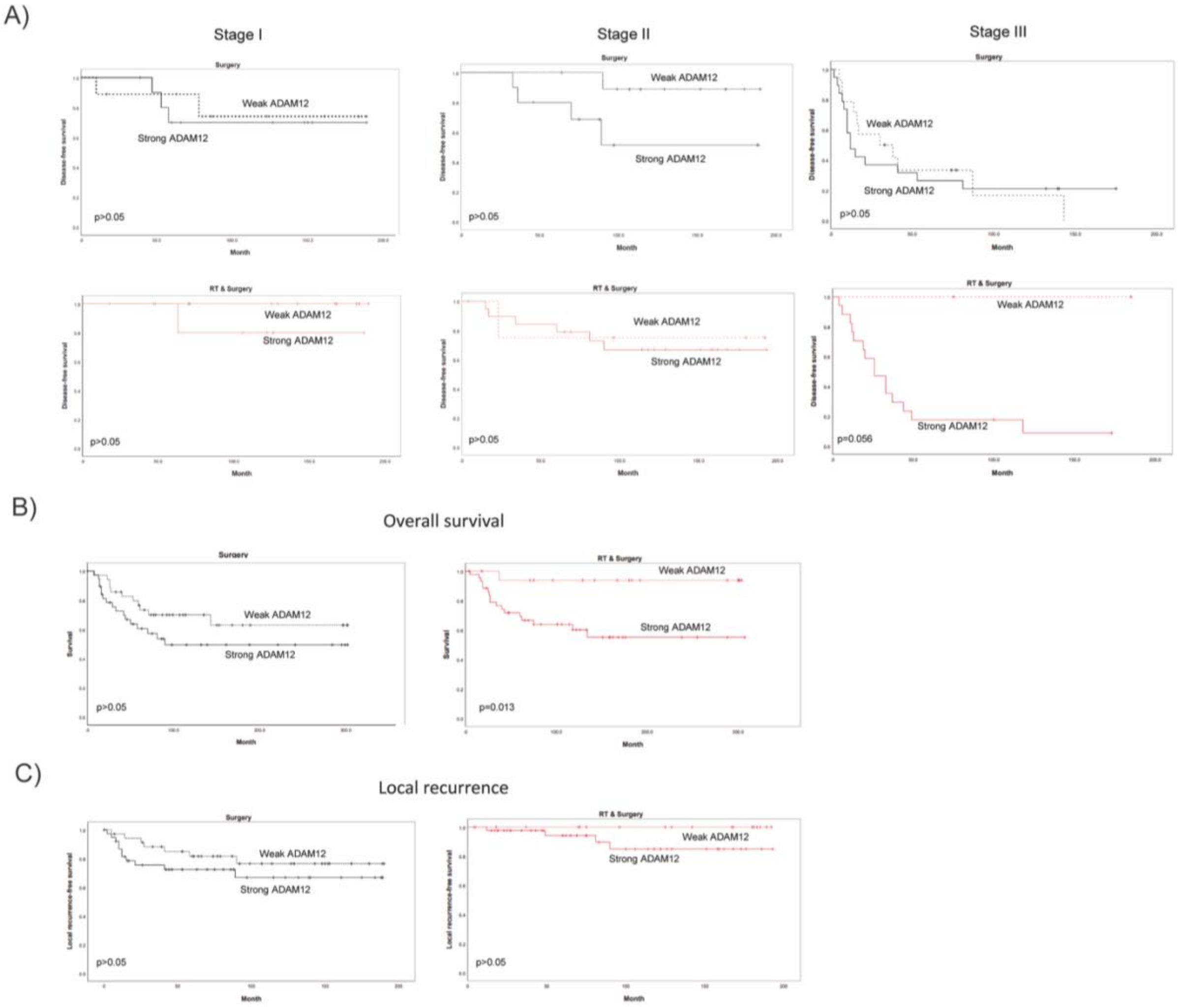

ADAM12 expression correlates with overall survival in rectal cancer patients following radiotherapy but not with local recurrence of the disease

Kaplan-Meier curves for A) disease-free survival of weak and strong ADAM12-expressing cases, separated into disease stages I, II, and III, B) overall survival, and C) local recurrence-free survival in the surgery alone and the preoperative radiotherapy plus surgery groups. Log-rank test was applied to test for significant differences.

Supplementary figure 2

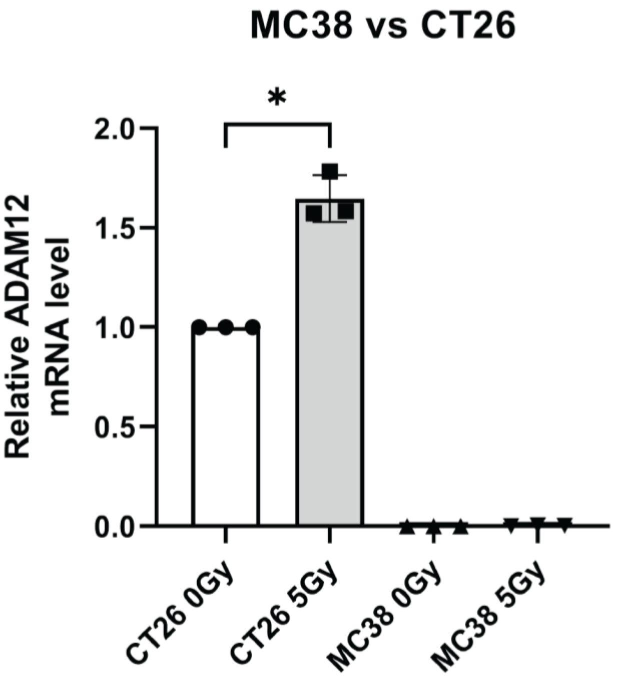

MC38 cells do not express ADAM12 in vitro with or without prior irradiation

Comparison of ADAM12 mRNA expression levels, quantified by qPCR, in wildtype CT26 and 4 MC38 cells 15h after irradiation with 0Gy or 5Gy (n=3). Repeated measures one-way ANOVA with 5 geisser-greenhouse correction and with correction for multiple comparisons using FDR control by 6 Benjamini, Krieger and Yekutieli method was applied to test for significant differences: * $p \leq 0.05$.

Supplementary figure 3

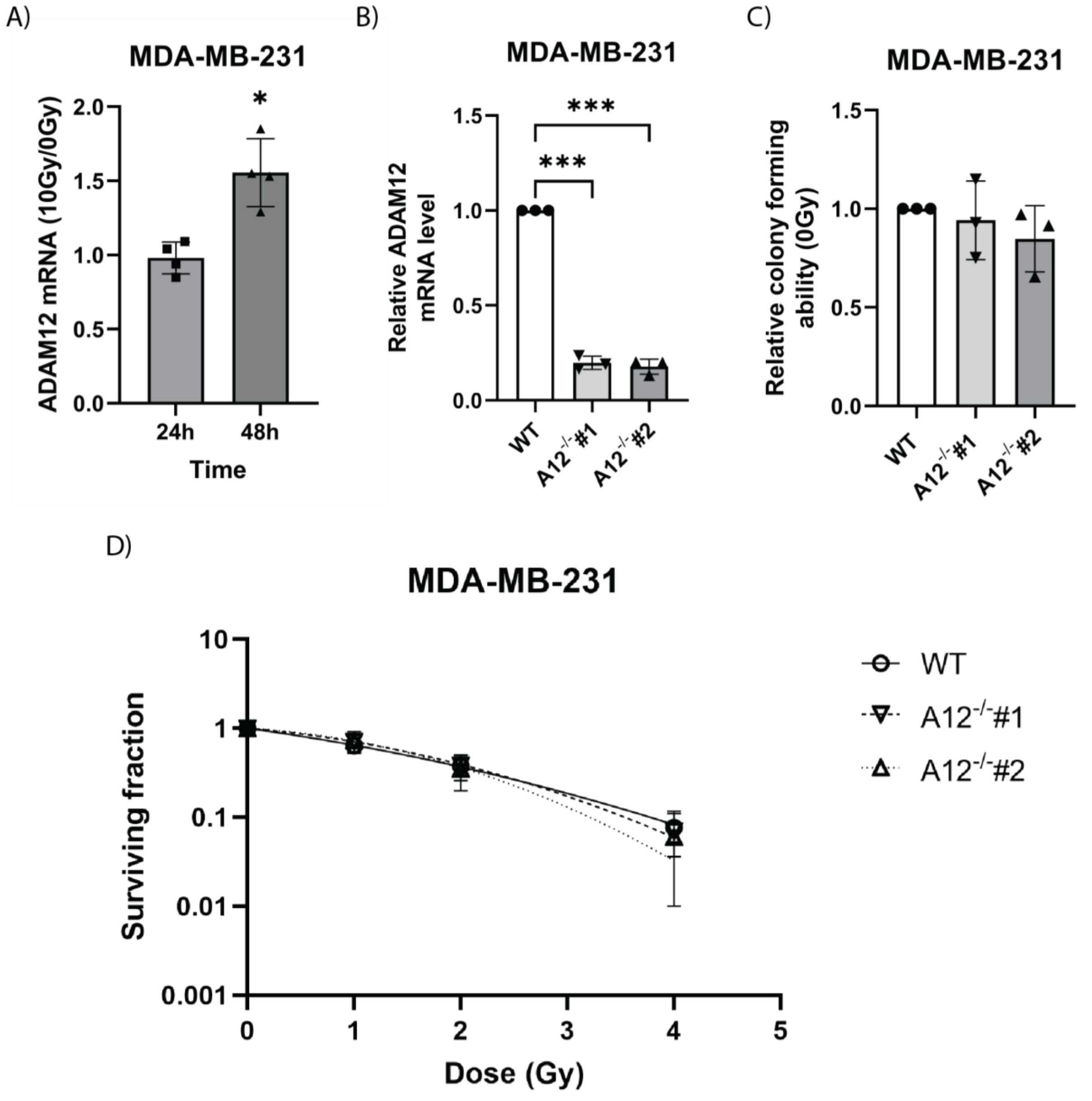

ADAM12 expression in MDA-MB-231 breast cancer cells is upregulated following IR and does not affect radiosensitivity or ability to form colonies

A) Fold change of ADAM12 mRNA levels in MDA-MB-231 human breast carcinoma cell line 10 following irradiation with 10Gy compared to 0Gy (n=4). B) qPCR validation of ADAM12 knockout 11 in MDA-MB-231 cells. C) MDA-MB-231 cell ability to form colonies following

loss of ADAM12 expression. D) Clonogenic assay of MDA-MB-231 wildtype (WT) and ADAM12-knockout (A12-/-) cells following irradiation. Repeated measures one-way ANOVA with correction for multiple comparisons using FDR control by Benjamini, Krieger and Yekutieli method (A-C) or extra sum-of-squares F-test of linear quadratic cell death models (D) was applied to test for significant differences: * $p \leq 0.05$, ** $p \leq 0.01$, *** $p \leq 0.001$. n=3 unless otherwise specified.

Supplementary table 1: qPCR and IDAA PCR primers list

**NAME**	**SEQUENCE**
qPCR_mouse_Adam12_Fwd	5'-TGTGGAAATGGCTATGTGGA-3'
qPCR_mouse_Adam12_Rev	5'-CAGGTGGTAGCGTTACAGCA-3'
qPCR_mouse_Gapdh_Fwd	5'-TGTTCCTACCCCCAATGTGT-3'
qPCR_mouse_Gapdh_Rev	5'-TGTGAGGGAGATGCTCAGTG-3'
qPCR_mouse_Rpl13a_Fwd	5'-ATGACAAGAAAAAGCGGATG-3'
qPCR_mouse_Rpl13a_Rev	5'-CTTTTCTGCCTGTTTCCGTA-3'
qPCR_mouse_B2m_Fwd	5'-ATTCACCCCCACTGAGACTG-3'
qPCR_mouse_B2m_Rev	5'-TGCTATTTCTTTCTGCGTGC-3'
qPCR_human_Adam12_Fwd	5'-TCAGCACGTGTTCTGGTCTC-3'
qPCR_human_Adam12_Rev	5'-CAGCGAGGTTTGGTGTGTTG-3'
qPCR_human_Gapdh_Fwd	5'-GCTGAGTACGTCGTGGAGTC-3'
qPCR_human_Gapdh_Rev	5'-GGTGCTAAGCAGTTGGTGGT-3'
qPCR_human_Rps9_Fwd	5'-TGTAACCAGAGACGTGATTGGC-3'
qPCR_human_Rps9_Rev	5'-TAGCAGCCGAATGAACTGCAT-3'
qPCR_human_Act3_Fwd	5'-GAAGAGCTACGAGCTGCCTG-3'
qPCR_human_Act3_Rev	5'-CGGATGTCCACGTCACACTT-3'
IDAA_mouse_Adam12_Fwd	5'-TCACCATAAATGAGGTACTGTCC-3'
IDAA_mouse_Adam12_Rev	5'-CAGCTCTCGGCTCAAGAAAC-3'
IDAA_human_Adam12_Fwd	5'-GGGCTATTGTTCCGA-3'
IDAA_human_Adam12_Rev	5'-AACGCCTACCACTAAGGGAC-3'
